# Topical Administration of 15-Deoxy-*Δ*^12,14^-Prostaglandin J_2_ Using a Nonionic Cream: Effect on UVB-Induced Skin Oxidative, Inflammatory, and Histopathological Modifications in Mice

**DOI:** 10.1155/2021/9330596

**Published:** 2021-11-02

**Authors:** Clovis M. Kumagai, Renata M. Martinez, Barbara B. Colombo, Priscila Saito, Ingrid C. Pinto, Camilla C. A. Rodrigues, David L. Vale, Ricardo L. N. Matos, Ana P. F. R. L. Bracarense, Marcela M. Baracat, Sandra R. Georgetti, Juliana T. Clemente-Napimoga, Marcelo H. Napimoga, Victor Fattori, Waldiceu A. Verri, Rúbia Casagrande

**Affiliations:** ^1^Departamento de Ciências Farmacêuticas, Universidade Estadual de Londrina, Avenida Robert Koch, 60, Hospital Universitário, 86039-440 Londrina Paraná, Brazil; ^2^Departamento de Ciências Patológicas, Universidade Estadual de Londrina, Rodovia Celso Garcia Cid, Km 80, PR445, Cx. Postal 10.011, 86057-970 Londrina, Brazil; ^3^Laboratório de Patologia Animal, Universidade Estadual de Londrina, Campus Universitário, Rodovia Celso Garcia Cid, Km 380, Londrina Paraná 86057-970, Brazil; ^4^Faculdade São Leopoldo Mandic, Instituto São Leopoldo Mandic, Laboratory of Neuro-Immune Interface of Pain Research, R. José Rocha Junqueira, 13, Campinas, São Paulo 13045-755, Brazil; ^5^Vascular Biology Program, Boston Children's Hospital, Department of Surgery, Harvard Medical School, Boston, MA, USA

## Abstract

UVB radiation is certainly one of the most important environmental threats to which we are subjected to. This fact highlights the crucial protective role of the skin. However, the skin itself may not be capable of protecting against UVB depending on irradiation intensity and time of exposition. Sun blockers are used to protect our skin, but they fail to fully protect it against oxidative and inflammatory injuries initiated by UVB. To solve this issue, topical administration of active molecules is an option. 15-Deoxy-*Δ*^12,14^-prostaglandin J_2_ (15d-PGJ_2_) is an arachidonic acid-derived lipid with proresolution and anti-inflammatory actions. However, as far as we are aware, there is no evidence of its therapeutic use in a topical formulation to treat the deleterious events initiated by UVB, which was the aim of the present study. We used a nonionic cream to vehiculate 15d-PGJ_2_ (30, 90, and 300 ng/mouse) (TFcPGJ_2_) in the skin of hairless mice. UVB increased skin edema, myeloperoxidase activity, metalloproteinase-9 activity, lipid peroxidation, superoxide anion production, gp91^phox^ and COX-2 mRNA expression, cytokine production, sunburn and mast cells, thickening of the epidermis, and collagen degradation. UVB also diminished skin ability to reduce iron and scavenge free radicals, reduced glutathione (GSH), sulfhydryl proteins, and catalase activity. TFcPGJ_2_ inhibited all these pathological alterations in the skin caused by UVB. No activity was observed with the unloaded topical formulation. The protective outcome of TFcPGJ_2_ indicates it is a promising therapeutic approach against cutaneous inflammatory and oxidative pathological alterations.

## 1. Introduction

15-Deoxy-*Δ*^12,14^-prostaglandin J_2_ (15d-PGJ_2_) is a cyclopentenone prostaglandin formed upon the spontaneous dehydration of prostaglandin D_2_ into 15d-PGJ_2_ [1]. Most of the studies on 15d-PGJ_2_ in the skin describe this prostaglandin as a protective molecule. In models used to study cancer, 15d-PGJ_2_ reduces the growth of the human melanoma cell strains (C8161, C8146C, and C8146A), human melanoma cell line (M1RW5) [2], PAM 212 transformed mouse epidermal cell line [3], and transformed human epidermal cell line (HSC-1) [4]. These results suggest the potential therapeutic application of 15d-PGJ_2_ as an anticancer molecule, but also it would not be a promoter of tumorigenesis, which is an important characteristic.

In terms of anti-inflammatory response, a topical poloxamer hydrogel containing 15d-PGJ_2_ reduced atopic dermatitis-like disease caused by topical administration of 2,4-dinitrochlorobenzene in mice. The mechanisms of 15d-PGJ_2_ hydrogel involved the reduction of immunostaining of ROR-*γ*t and TNF-*α*, which suggest a reduction of adaptive immunity with reduced Th17 lymphocytes [5]. Opposing to these data, it was found that 15d-PGJ_2_ can work as a neuritogenic promoter by activating TRPV1 channels and enhancing NGF activity, which ultimately aggravates atopic dermatitis [6]. Thus, there is controversy on the biological activities of 15d-PGJ_2_ in atopic dermatitis. Further expanding the biological activities of 15d-PGJ_2_ and possible routes of administration, microneedles enhanced the topical delivery of that prostaglandin increasing its analgesic activity against the formalin-induced temporomandibular joint pain. The 15d-PGJ_2_ microneedle mechanism involved the reduction of TNF-*α* and IL-1*β* release [7]. These are inflammatory cytokines [8].

UVB irradiation causes skin inflammation, which is dependent on the production of cytokines and activation of tissue-resident cells and recruitment of leukocytes [9]. Adding to this inflammatory milieu, oxidative stress is an essential mechanism of UVB pathological alterations in the skin [10]. Cytokines and reactive oxygen species (ROS) play crucial roles in activating mast cells, keratinocytes, and resident macrophages. These cells release additional batches of inflammatory cytokines and produce ROS, which recruit leukocytes. Neutrophils are the main leukocytes recruited in acute UVB irradiation challenge in the skin [11]. At this stage, keratinocyte apoptosis leads to the formation of sunburn cells, and epidermal proliferation and plasma exudation cause the thickening of the epidermis [9]. Thus, to prevent the pathological alterations caused by UVB irradiation in the skin, the inflammatory and/or oxidative processes must be stopped to prevent chronic outcomes such as carcinogenesis.

The literature mentioned above makes clear that there is still controversy whether 15d-PGJ_2_ is a treatment or aggravates atopic dermatitis, which is an inflammatory skin disease. This is obviously a difficult question to answer, which is related to the varied experimental conditions that each study used in terms of the route of administration, dose/concentration, disease model, and timeframe of disease in which 15d-PGJ_2_ was administrated. However, despite these data, no study investigated the effect of 15d-PGJ_2_ topical treatment against the pathological alterations caused by UVB irradiation. Thus, this was the aim of our study and we found that topical treatment with 15d-PGJ_2_ reduced the inflammatory and oxidative pathological modifications caused by UVB irradiation in the skin of hairless mice.

## 2. Materials and Methods

### 2.1. Materials

15d-PGJ_2_ was from Cayman Chemical (Ann Arbor, Michigan, EUA). *tert*-Butyl hydroperoxide was from Acros Organics (Geel, Antwerp, Belgium). Brilliant Blue R, reduced glutathione (GSH), hexadecyltrimethylammonium bromide (HTAB), N-ethylmaleimide, *o*-dianisidine dihydrochloride, phenylmethanesulfonyl fluoride, 5,5′-dithiobis(2-nitrobenzoic acid) (DTNB), nitroblue tetrazolium (NBT), 2,2′-azino-bis(3-ethylbenzothiazoline-6-sulfonic acid) (ABTS), Trolox, TPTZ (2,4,6-tris(2-pyridyl)-s-triazine), and bisacrylamide were obtained from Sigma-Aldrich (St. Louis, MO, USA). Xylene cyanol, acrylamide, sodium dodecyl sulfate (SDS), glycerol, and hydroxymethyl aminomethane (Tris) were obtained from Amresco (Solon, OH, USA). ELISA kits for the determination of cytokines were obtained from eBioscience (San Diego, CA, USA). Reverse transcriptase (SuperScript® III), Oligo(dT)12-18 primers, Platinum SYBR Green®, and primers were from Invitrogen (Carlsbad, CA, USA). Isoflurane was from Abbott (Chicago, IL, USA). The excipients used for the preparation of the formulation (Polawax®, caprylic/capric triglyceride, and Phenonip®) were obtained from Galena (Campinas, SP, Brazil). All other reagents used were of pharmaceutical grade.

### 2.2. Topical Formulation Containing 15d-PGJ_2_

The formulation was prepared using the following: (i) the self-emulsifying wax Polawax® (cetostearyl alcohol and polyoxyethylene derived of a fatty acid ester of sorbitan 20 0E) (10%); (ii) the emollient caprylic/capric triglyceride (5%); (iii) the solubilizing agent and moisturizer propylene glycol (6%); (iv) the preservative Phenonip (1%); and (v) deionized water to complete 100% of the formulation. 15d-PGJ_2_ was incorporated into the topical formulation in different concentrations. The control unloaded topical formulation was named uTF; topical formulation containing 15d-PGJ_2_ was named TFcPGJ_2_.

### 2.3. Therapeutic Effect of Topical Treatment with 15d-PGJ_2_ in Photooxidative and Inflammatory Skin Damage Triggered by UVB Irradiation

#### 2.3.1. Animals

The experiments were performed using hairless mice (HRS/J), weighing 25-30 g, produced by the Animal House of the University Hospital of Londrina State University. Mice had free access to water and food at a temperature of 23°C ± 2 and a 12 h light and 12 h dark cycles. The Animal Ethics Committee (Of. Circ. CEUA-UEL no. 017/2015, process no. 1447.2015.10) of Londrina State University approved all procedures. All efforts were made to minimize animal use and their suffering.

#### 2.3.2. Experimental Protocol

**Figure 1:**
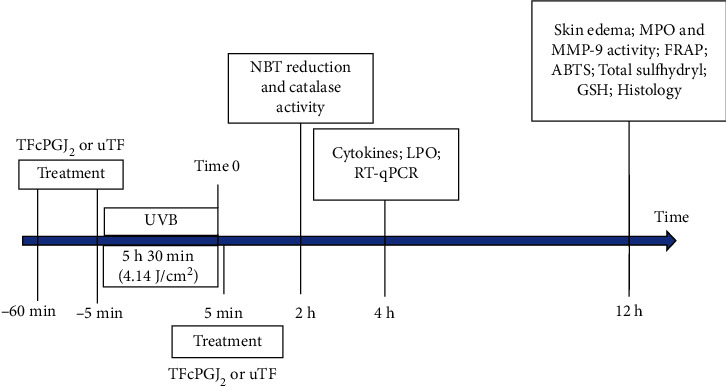
Schematic protocol approaching the treatment with topical formulation containing 15d-PGJ_2_ (TFcPGJ_2_), time points of sample collection, and parameters analyzed at each time point. Mice were treated with TFcPGJ_2_ or unloaded topical formulation (uTF) 1 h and 5 min before the beginning of irradiation and 5 min after the irradiation. Dorsal skin samples were collected 12 h after the exposure to UVB irradiation for skin edema, MPO and MMP-9 activity, FRAP, ABTS, total sulfhydryl, GSH levels, and histology. Samples were collected after 2 hours of exposure to UVB for the tests of catalase and superoxide anion production (NBT reduction). Finally, samples collected 4 hours after the end of the irradiation were tested for cytokines and lipid peroxidation (LPO) and mRNA expression by RT-qPCR.

ARRIVE guidelines were followed.  Figure 1 shows that three time points of sample collection were used. At each time point, samples of the same mice were collected for the indicated methods. The first time point used to assess the activity of topical formulation containing 15d-PGJ_2_ (TFcPGJ_2_) was 12 h in which a dose-response curve was performed to evaluate skin edema, myeloperoxidase activity, metalloproteinase-9 activity, and antioxidant markers. These data were used to select one dose of TFcPGJ_2_ (300 ng/mouse) to continue with the histological processing of samples collected at 12 h from the same mice. The dose of 300 ng of 15d-PGJ_2_ per mouse was also used at 2 h (tests: nitroblue tetrazolium reduction and catalase activity) and 4 h (tests: lipid peroxidation, cytokines, and RT-qPCR) time points as well. A total of 168 HRS/J mice were randomly assigned to different groups with 6 mice each. Every experiment was performed twice. The groups were the nonirradiated control group (NC (negative control)), irradiated control group (PC (positive control)), irradiated group treated (three times) with unloaded topical formulation (uTF—a vehicle control), and three irradiated groups treated with TFcPGJ_2_. The amount of 0.5 g of the formulation was applied on the dorsal surface skin. The TFcPGJ_2_ contained graded concentrations of 15d-PGJ_2_ (10, 30, and 100 ng/0.5 g of the formulation), and application was performed three times (at 1 h before, at 5 min before, and at 6 h after the beginning of the UVB irradiation session) generating the final cumulative doses of 30, 90, and 300 ng/mouse of TFcPGJ_2_. The doses of 15d-PGJ_2_ used in treatments were selected based on the therapeutic effects of previously published studies in other disease models [12].

#### 2.3.3. Irradiation

The light source used in the experiments to induce oxidative stress and acute inflammatory processes in hairless mice was a fluorescent UVB lamp model PHILIPS TL/12 40W RS (MEDICAL-NETHERLANDS). The lamp emits radiation at a wavelength (*ƛ*) between 270 and 400 nm with a maximum emission peak around 313 nm. The radiation dose used to induce inflammation and oxidative stress was 4.14 J/cm^2^ for a period of five hours and thirty minutes [13]. The lamp is attached at the top, in a rectangular wooden box with a capacity of 6 boxes, used only for experiments with this purpose. To measure the irradiation of the lamp, an IL1700 radiometer device was used to detect UVB (SED240) radiation [14]. The mice were terminally anesthetized with 5% isoflurane 12 h or anesthetized followed by decapitation at 2 h or 4 h after UVB exposure, and afterwards, dorsal skin samples were collected followed by storage at -80°C before analysis. The exception was the cutaneous edema in which samples were weighed immediately after collecting and the histology in which samples were fixed in buffered formaldehyde. Each parameter was evaluated at a specific time point according to the prior determination of suitability to detect significant differences between the negative and positive control groups and, therefore, being suitable for the determination of possible treatment effects [15].

#### 2.3.4. Skin Edema

For this test, samples of the animals' dorsal skin were collected using a mold with a fixed area (5 mm in diameter). Edema was measured by the skin weight of this fixed area, and the mean weight of each group was compared to every other group. The results were expressed as skin weight (mg) [16].

#### 2.3.5. Activity of the Enzyme Myeloperoxidase as a Surrogate of Neutrophil Counts

Neutrophil recruitment was indirectly assessed by the activity of the myeloperoxidase (MPO) enzyme, which was executed under the following procedures: the sample of skin was homogenized in 0.05 M phosphate buffer (pH 6.0) containing 0.5% hexadecyltrimethylammonium bromide (HTAB). The homogenates were centrifuged for 2 min under refrigeration (4°C) at 11,000 rpm, and after this step, 30 *μ*L of the resulting supernatant was diluted in 200 *μ*L of reaction solution consisting of phosphate buffer (0.05 M; pH 6.0) with *o*-dianisidine dihydrochloride (0.0167%) and hydrogen peroxide (0.05%). Upon adding all reagents mentioned, a period of 20 min was allowed for the reaction. A titrated curve of neutrophils was used for the comparison of MPO activity. Readings in spectrophotometry were at 450 nm. The results are presented as MPO activity (number of neutrophils per milligram of skin) [17].

#### 2.3.6. Analyses of Skin Proteinase Substrate-Embedded Enzymography

For the assay to demonstrate the activity of MMP-9, the technique of polyacrylamide gel zymography with sodium dodecyl sulfate (SDS) was used. The analysis detects gelatin-degrading enzymes present in the gel [18]. Skin samples from each group were pooled and stored in vials. The samples were homogenized with the aid of the tissue homogenizer, in a 1 : 4 ratio in phosphate buffer Tris/HCl 50 mM (pH 7.4) with calcium chloride and 1% of proteinase inhibitors. The homogenates were centrifuged, and the supernatants were included in the zymography assay. An aliquot of 50 *μ*L of the supernatant was added to 10 *μ*L of Tris/HCl buffer (pH 6.8) containing 20% glycerol, 4% sodium dodecyl sulfate (SDS), and 0.001% bromophenol blue. After homogenization, the samples were placed in a water bath at 37°C for 8 minutes immediately before being applied to the electrophoresis gel containing 0.025% gelatin and 10% acrylamide. After the electrophoresis procedure, the gels were incubated for 1 h with 2.5% Triton X-100 under constant shaking, incubated overnight in 0.05 M Tris-HCl (pH 7.4), 0.01 M CaCl_2_, and 0.02% sodium azide at 37°C, and stained the following day with Brilliant Blue R. After destaining by a 20% acetic acid solution, the zone of enzyme activity was analyzed by comparing the groups in the ImageJ program (NIH, Bethesda, MD, USA) [15].

#### 2.3.7. Total Antioxidant Capacity: FRAP Assay

The FRAP test is a colorimetric method that measures the iron reduction of TPTZ, a colored product formed by the reaction of antioxidants that donates electrons in the process [19]. The test was adapted and used to assess the antioxidant power in skin samples [17]. The skin samples were homogenized in 400 *μ*L of KCl (1.15%) and centrifuged at 1,000 g for 10 min at 4°C. For the reaction, 30 *μ*L of the sample homogenate supernatant was used. The FRAP reagent was prepared by adding 2.5 mL of a 10 mM solution of TPTZ in 40 mM HCl with 2.5 mL of 20 mM iron chloride hexahydrate and 25 mL of 0.3 mM acetate buffer (pH 3.6), and this solution was incubated at 37°C for 30 min before use. Then, the reading was performed at 595 nm. Standard solutions with different concentrations of Trolox (0.5 to 20 *μ*M) (a synthetic analog of the antioxidant vitamin E) were used for calibration. Results were expressed as *μ*M equivalent of Trolox/mg skin [19].

#### 2.3.8. Total Antioxidant Capacity: ABTS Assay

The antioxidant capacity of each sample was measured by the decay of the coloration of the ABTS+ cation radical when electrons are donated to it by the antioxidant. For the ABTS reaction, the samples were homogenized in 400 *μ*L of 1.15% KCl using the tissue homogenizer and centrifuged at 1,000 g for 10 min at 4°C; then, the supernatant was used for analysis. The ABTS solution was prepared after reacting 7 mM of the ABTS solution with 2.45 mM of potassium persulfate resulting in the ABTS+ cation. The mixture was stored in an amber bottle for at least 16 hours before use. After, the ABTS solution was mixed with phosphate buffer until it reached an absorbance of 0.8 at 730 nm. The supernatant was mixed with the ABTS solution, and after 6 min, the absorbance was determined at 730 nm. A standard curve was prepared with titrated Trolox concentrations (1 to 25 *μ*M), and the results were expressed as *μ*M equivalent of Trolox/mg skin [17, 19].

#### 2.3.9. Quantification of Endogenous Antioxidant Reduced Glutathione (GSH) and Total Sulfhydryl Groups

Briefly, the skin samples were homogenized in 0.02 M EDTA. In the GSH assay, the homogenates were treated with 50% trichloroacetic acid and centrifuged at 2,700 g for 10 min at 4°C. In the total sulfhydryl group assay, the homogenates were centrifuged at 2,700 g for 10 min at 4°C. The supernatant was separated and centrifuged again at 2,700 g for 15 min at 4°C. Fifty *μ*L of the final supernatant was mixed with 100 *μ*L of 0.4 M Tris buffer pH 8.9 and 5 *μ*L of a 1.9 mg/mL solution of DTNB in methanol. Reading was performed at 405 nm, and calculations were relative to a GSH standard curve (5–150 *μ*M). The results were expressed as *μ*M of GSH or of total sulfhydryls per mg of skin [9, 20].

#### 2.3.10. Catalase Activity Assay

The catalase activity is based on measuring the decay of hydrogen peroxide (H_2_O_2_) concentration. Reading was performed at 240 nm at 25°C, and catalase activity was calculated based on the difference between the reading before and 30 sec after the addition of H_2_O_2_. The values of catalase concentration were expressed as a unit of catalase/mg of skin/minute [9, 21].

#### 2.3.11. Assay for Lipid Peroxidation (LPO)

The hydroperoxide production was evaluated by the chemiluminescence method previously described [20]. Skin samples were homogenized in 800 *μ*L of phosphate buffer (pH 7.4) and centrifuged (700 g, 2 min, 4°C). In the second step, 250 *μ*L of the supernatant was added to 1,730 *μ*L of reaction medium (120 mM KCl, 30 mM phosphate buffer pH 7.4) and 20 *μ*L of 3 mM *tert*-butyl hydroperoxide. The reading was conducted in a *b*-counter Beckman R LS 6000SC in a noncoincident counting for 30 s with a response range between 300 and 620 nm. All runs last 120 min (30°C). The results were measured in counts per min (cpm) per mg of skin tissue.

#### 2.3.12. Superoxide Anion Production

Skin samples were homogenized in 0.02 M EDTA and centrifuged at 2,000 g, for 20 seconds at 4°C. In the second step, the supernatant, 50 *μ*L, was incubated in a 96-well plate for 1 h. The nonadherent/nonprecipitated supernatant was carefully removed, followed by the addition of 100 *μ*L of NBT (1 mg/mL) to each well, and incubated for 15 min. The NBT reagent was then carefully removed and followed by the addition of 20 *μ*L methanol 100%. Finally, the formazan, a compound formed by the reduction of NBT, was solubilized by adding 120 *μ*L of KOH 2 M and 140 *μ*L of dimethylsulfoxide. Then, the reading was performed at 620 nm. Results are expressed as NBT reduction (OD/10 mg of skin) [20, 22].

#### 2.3.13. Reverse Transcriptase (RT) and Quantitative Polymerase Chain Reaction (qPCR)

Skin samples were homogenized in the TRIzol® reagent, and total RNA was isolated according to the manufacturer's directions [9]. RT-qPCR was performed using the GoTaq® 2-Step RT-qPCR System (Promega) on a StepOnePlus™ Real-Time PCR System (Applied Biosystems®). The relative gene expression was measured using the comparative 2^-(∆∆Ct)^ method. GAPDH was used as a housekeeping gene. Primer sequences are shown in Table 1.

#### 2.3.14. Cytokine Measurements

The skin levels of the cytokines TGF-*β* and IL-6 were measured using commercial enzyme-linked immunosorbent assay (ELISA) kits according to the manufacturer's instructions (eBioscience). For that, the dorsal skin samples were homogenized in 500 *μ*L sterile saline and centrifuged (2,000 g, 15 min, 4°C). The supernatants were used to determine cytokine levels. Reading was performed at 450 nm in a microplate spectrophotometer reader. The results were obtained by comparing the optical densities of the samples with the densities of the respective cytokine standard curves [23].

#### 2.3.15. Skin Histologic Evaluation

The skin samples were fixed in 10% buffered formaldehyde solution, embedded in paraffin, and sectioned (5 *μ*m). The sections were stained with Masson's trichrome staining for collagen fiber analysis. Degradation of collagen fibers was analyzed by the intensity of the blue coloration in the dermal areas of the skin using light microscopy (10x magnification). For the determination of epidermal thickness [24] and for counting the number of sunburn cells [25], tissue sections were stained with hematoxylin and eosin (H&E) and analyzed using light microscopy at a magnification of 40x and 100x, respectively. Toluidine blue staining was also used to determine mast cell counts (40x magnification) [26]. Analyses were done with the software Infinity Analyze (Lumenera® Software) [20].

### 2.4. Statistical Analysis

Statistical analysis was performed using GraphPad Prism 7 software (GraphPad Software Inc., San Diego, USA). Data were analyzed by one-way analysis of variance (ANOVA) followed by Tukey's multiple comparison test. Results were presented as mean ± standard error (SEM) of measurements made with 6 animals in each group per experiment. The results are representative of 2 separate experiments and were considered significantly different at *p* < 0.05.

## 3. Results

### 3.1. Topical Formulation Containing 15d-PGJ_2_ (TFcPGJ_2_) Reduced the Skin Edema, MPO Activity, and MMP-9 Activity/Secretion Increase Caused by UVB Irradiation

**Figure 2:**
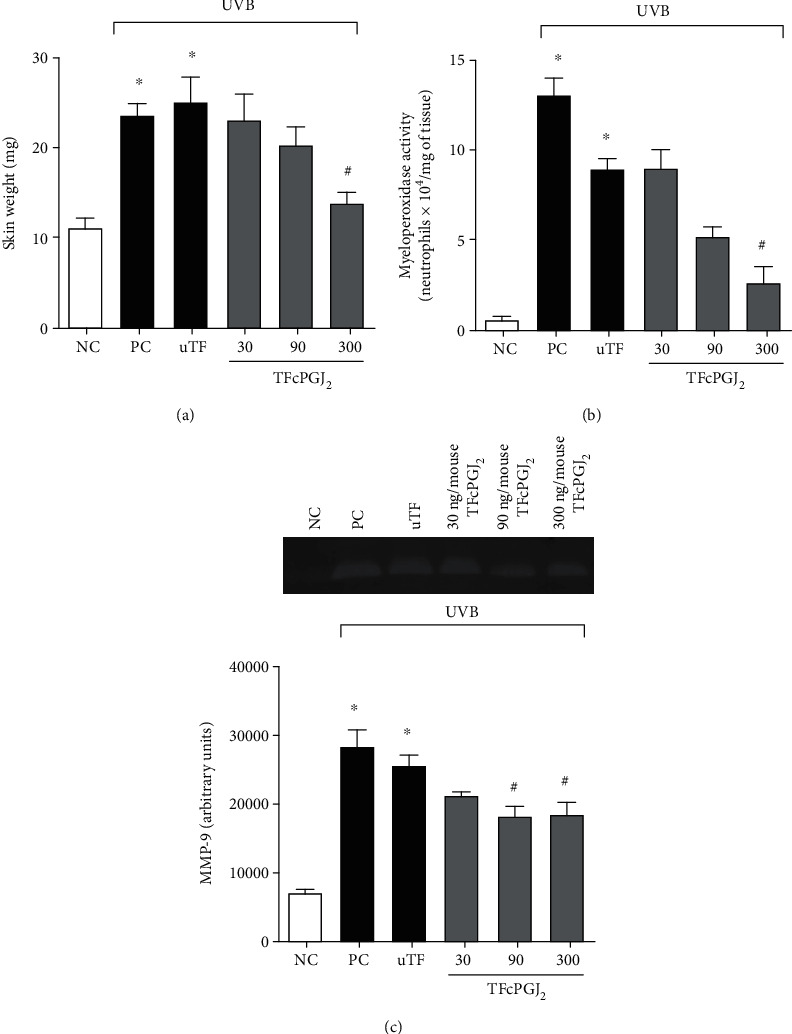
Topical formulation containing 15d-PGJ_2_ (TFcPGJ_2_) inhibits skin edema, myeloperoxidase activity, and MMP-9 activity triggered by UVB. The skin edema (a), myeloperoxidase activity (b), and MMP-9 activity (c) were determined in samples collected 12 h after the end of irradiation. Panel (c) presents data analysis of the activity of MMP-9 and a representative image. NC: negative control; PC: positive control; uTF: unloaded topical formulation. Bars are means ± SEM of 6 mice per group and are representative of two separate experiments. One-way ANOVA followed by Tukey's test. ^∗^*p* < 0.05 versus the negative control group; ^#^*p* < 0.05 versus UVB-stimulated groups.

In all experiments, we used negative and positive control groups to confirm the induction of response by UVB irradiation compared to naive mice. An unloaded topical formulation (uTF) and TFcPGJ_2_ were also used.  Figure 1 presents the schematic protocol with treatment, time points of sample collection, and parameters analyzed at each time point according to prior publications [9, 13–15, 17, 18, 20–24]. The first set of experiments evaluated whether TFcPGJ_2_ would inhibit skin inflammation in a dose-dependent manner (30, 90, and 300 ng/animal) having UVB irradiation as the stimulus (Figure 2). Skin edema was reduced only by the dose of 300 ng/animal of TFcPGJ_2_ (Figure 2(a)). In the MPO activity assay, again, 300 ng/animal of TFcPGJ_2_ was effective in reducing this inflammatory parameter (Figure 2(b)). Despite not being significant, the effect of 90 ng/animal of TFcPGJ_2_ presented a *p* = 0.2191 compared to the unloaded topical formulation and was not statistically different compared to the dose of 300 ng/animal of TFcPGJ_2_. In agreement with this trend, both 90 and 300 g/animal of TFcPGJ_2_ significantly inhibited the inflammatory activity of MMP-9 compared to the uTF (Figure 2(c)). In all these three assays, we observed that UVB irradiation induced a significant response compared to the naive negative control group. There was no statistical difference comparing the UVB positive control group and the UVB irradiation plus uTF, which indicates that the uTF was not active per se (Figure 2).

### 3.2. TFcPGJ_2_ Reduces Antioxidant Depletion Caused by UVB Irradiation

**Figure 3:**
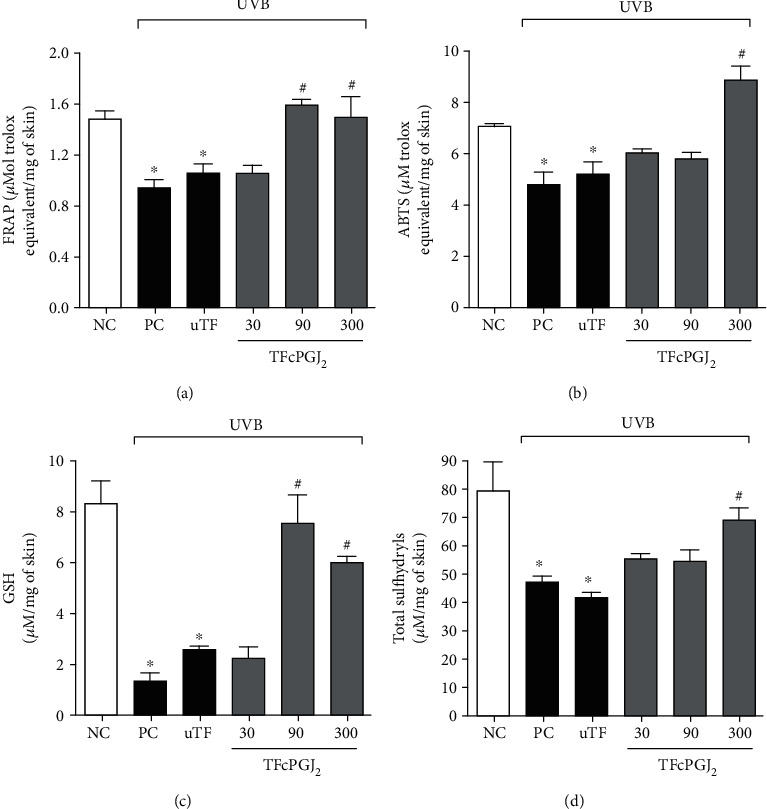
Topical formulation containing 15d-PGJ_2_ (TFcPGJ_2_) inhibits the depletion of endogenous antioxidants by UVB irradiation. The antioxidant capacity was measured using FRAP (a), ABTS (b), GSH (c), and total sulfhydryl (d) assays in samples collected 12 h after the end of irradiation. NC: negative control; PC: positive control; uTF: unloaded topical formulation. Bars are means ± SEM of 6 mice per group and are representative of two separate experiments. One-way ANOVA followed by Tukey's test. ^∗^*p* < 0.05 versus the negative control group; ^#^*p* < 0.05 versus UVB-stimulated groups.

In this set of analyses, four antioxidant parameters were evaluated: the ability of the skin's endogenous antioxidants to reduce iron (FRAP; Figure 3(a)) and scavenge the synthetic cationic radical ABTS (Figure 3(b)); the levels of reduced glutathione (GSH) (Figure 3(c)) that is an antioxidant tripeptide; and the total sulfhydryl groups representing antioxidants containing the thiol chemical group (Figure 3(d)). The dose of 300 ng/animal of TFcPGJ_2_ dampened the reduction of FRAP (Figure 3(a)), ABTS (Figure 3(b)), GSH levels (Figure 3(c)), and total sulfhydryl groups (Figure 3(d)) caused by UVB irradiation compared to the uTF control group. The dose of 90 ng/animal of TFcPGJ_2_ was active against the depletion of FRAP activity (Figure 3(a)) and reduction of GSH levels (Figure 3(c)) caused by UVB irradiation compared to the uTF control group. In all these four assays, we observed that UVB irradiation induced a significant depletion of antioxidant responses compared to the naive negative control group. There was no statistical difference comparing the UVB positive control group and the UVB irradiation plus uTF, which indicates that the uTF was not active per se (Figure 3). Data presented in Figures 2 and 3 were obtained with samples collected at 12 h and showed that 300 ng/animal of TFcPGJ_2_ is the ideal dose of treatment; thus, it was selected for the following experiments.

### 3.3. TFcPGJ_2_ Prevents Catalase Activity Depletion and Inhibits the Formation of Lipid Peroxides and Superoxide Anion Production as well as Reduces the mRNA Expression of gp91^phox^ and Cyclooxygenase-2 (COX-2) Triggered by UVB Irradiation

**Figure 4:**
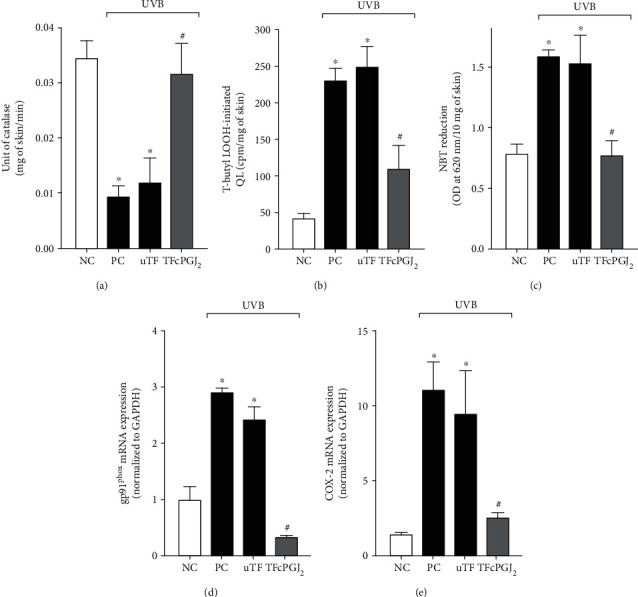
Topical formulation containing 15d-PGJ_2_ (TFcPGJ_2_) inhibits the depletion of endogenous catalase activity and the enhanced production of lipid peroxides and superoxide anion as well as reduces gp91^phox^ and cyclooxygenase (COX-2) mRNA expression in UVB-irradiated skin. Catalase activity (a) was determined in samples collected 2 h after the end of irradiation. Lipid peroxidation (b) was measured by a chemiluminescence (QL) assay initiated by *tert*-butyl hydroperoxide (LOOH) on samples collected 4 h after the end of irradiation. Superoxide anion production (c) was measured by the nitroblue tetrazolium (NBT) reduction test in samples collected 2 h after the end of irradiation. NADPH oxidase subunits gp91^phox^ (d) and COX-2 (e) were determined in samples collected 4 h after the end of irradiation by RT-qPCR. NC: negative control; PC: positive control; uTF: unloaded topical formulation. Bars are means ± SEM of 6 mice per group and are representative of two separate experiments. One-way ANOVA followed by Tukey's test. ^∗^*p* < 0.05 versus the negative control group; ^#^*p* < 0.05 versus UVB-stimulated groups.

Catalase converts superoxide anion into H_2_O and O_2_ and can be seen much more as an antioxidant pathway whose activity is depleted by excessive free radical formation [27]. In fact, UVB irradiation depleted the endogenous skin catalase activity compared to naive mouse skin. The UVB irradiation plus uTF presented a similar catalase activity compared to the UVB irradiation positive control group. Thus, the induction of catalase depletion by UVB irradiation worked properly and uTF had no activity per se (Figure 4(a)). On the other hand, TFcPGJ_2_ (300 ng/animal) inhibited the catalase activity depletion caused by UVB irradiation (Figure 4(a)). In agreement with the maintenance of catalase activity, TFcPGJ_2_ also inhibited the production of *tert*-butyl-initiated lipid peroxides (Figure 4(b)) and superoxide anion production (Figure 4(c)), which are an end product of oxidative stress and a reactive oxygen species, respectively. Two enzymes that link oxidative stress and inflammation are the phagocyte NADPH oxidase gp91^phox^ and prostanoid-producing enzyme COX-2 [28, 29]. UVB irradiation and UVB irradiation plus uTF presented similar induction of gp91^phox^ (Figure 4(d)) and COX-2 (Figure 4(e)) mRNA expression compared to the naive control group. TFcPGJ_2_ reduced the mRNA expression of gp91^phox^ (Figure 4(d)) and COX-2 (Figure 4(e)) compared to the UVB irradiation plus uTF control group.

### 3.4. TFcPGJ_2_ Reduces the Production of TGF-*β* and IL-6 Triggered by UVB Irradiation

**Figure 5:**
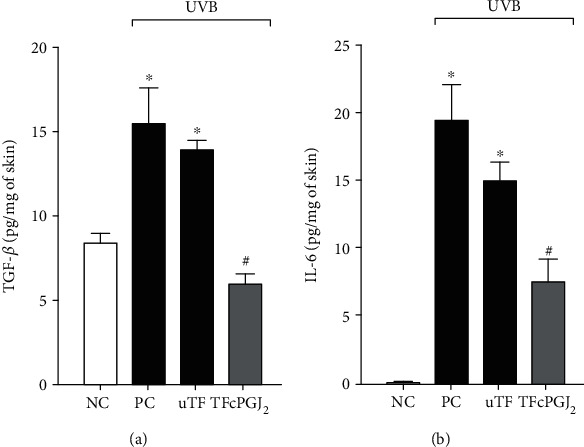
The UVB-triggered production of the cytokines was inhibited by topical formulation containing 15d-PGJ_2_ (TFcPGJ_2_). The cytokines TGF-*β* (a) and IL-6 (b) were determined in skin samples collected 4 h after the irradiation. NC: negative control; PC: positive control; uTF: unloaded topical formulation. Bars are means ± SEM of 6 mice per group and are representative of two separate experiments. One-way ANOVA followed by Tukey's test. ^∗^*p* < 0.05 versus the negative control group; ^#^*p* < 0.05 versus UVB-stimulated groups.

TGF-*β* is a cytokine involved in tissue repair and fibrosis. Its levels are increased when such responses are needed [30]. UVB irradiation with or without uTF caused an increase of TGF-*β* in the skin, likely because it causes tissue damage requiring tissue repair. TFcPGJ_2_ treatment reduced the production of TGF-*β* triggered by UVB irradiation (Figure 5(a)). IL-6, in turn, is a proinflammatory cytokine [31]. Again, UVB irradiation with or without uTF caused an increase of IL-6, which was inhibited by TFcPGJ_2_ treatment (Figure 5(b)).

### 3.5. TFcPGJ_2_ Reduces the Induction of Sunburn Cells Triggered by UVB

**Figure 6:**
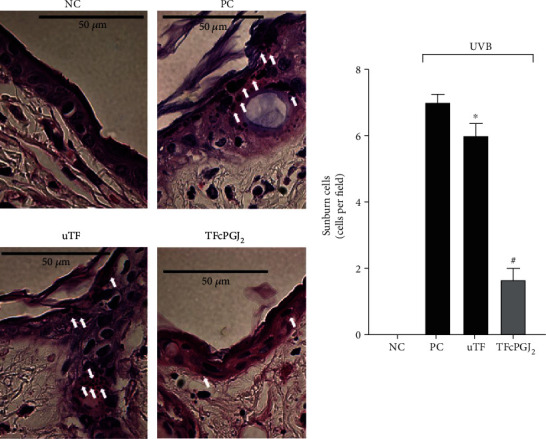
UVB irradiation triggers sunburn cell formation that is amenable by topical formulation containing 15d-PGJ_2_ (TFcPGJ_2_) treatment. Sunburn cell (white arrows) counts were evaluated using hematoxylin and eosin-stained slices of skin samples collected 12 h after the end of irradiation. Stained slices were examined using light microscopy at 100x magnification. NC: negative control; PC: positive control; uTF: unloaded topical formulation. Bars are means ± SEM of 6 mice per group and are representative of two separate experiments. One-way ANOVA followed by Tukey's test. ^∗^*p* < 0.05 versus the negative control group; ^#^*p* < 0.05 versus UVB-stimulated compared groups.

Sunburn cells are apoptotic keratinocytes with eosinophilic cytoplasm [32]. UVB irradiation induced sunburn cells compared to the nonirradiated control group. The uTF was inactive, and TFcPGJ_2_ reduced the induction of sunburn cells (Figure 6).

### 3.6. TFcPGJ_2_ Reduces Mast Cell Presence in the Dermis Recruited upon UVB Irradiation

**Figure 7:**
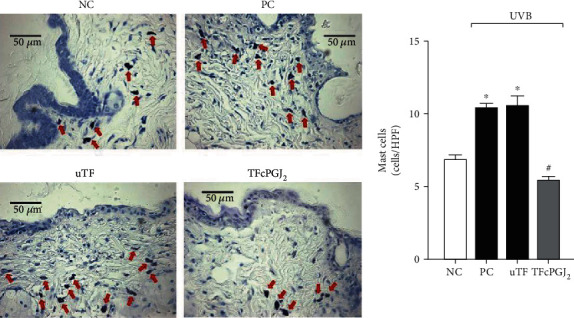
Topical formulation containing 15d-PGJ_2_ (TFcPGJ_2_) inhibits UVB irradiation-induced mast cell counts. Mast cell (red arrows) counts were evaluated using toluidine blue in skin samples collected 12 h after the irradiation and were examined using light microscopy at 40x magnification. NC: negative control; PC: positive control; uTF: unloaded topical formulation. Bars are means ± SEM of 6 mice per group and are representative of two separate experiments. One-way ANOVA followed by Tukey's test. ^∗^*p* < 0.05 versus the negative control group; ^#^*p* < 0.05 versus UVB-stimulated groups.

Mast cells are tissue-resident cells and one of the first cells to respond to inflammatory stimulation [33]. UVB irradiation induces the increase of mast cells in the dermis, since their recruitment occurs to amplify the inflammatory response [26]. In agreement with that, we observed that UVB irradiation successfully increased the number of mast cells in the dermis compared to the naive group and that the uTF was inactive (Figure 7). The TFcPGJ_2_ significantly reduced the number of mast cells recruited upon UVB irradiation stimulation (Figure 7).

### 3.7. TFcPGJ_2_ Reduces Collagen Fiber Degradation Triggered by UVB Irradiation

**Figure 8:**
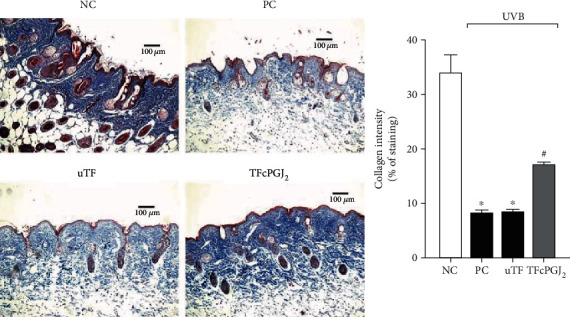
Topical formulation containing 15d-PGJ_2_ (TFcPGJ_2_) inhibits UVB irradiation-induced collagen fiber damage. Collagen fiber degradation was evaluated with Masson's trichrome staining in skin samples collected 12 h after the irradiation. Collagen fiber intensity and bundles shown in blue were examined by the ImageJ program (10x magnification). NC: negative control; PC: positive control; uTF: unloaded topical formulation. Bars are means ± SEM of 6 mice per group and are representative of two separate experiments. One-way ANOVA followed by Tukey's test. ^∗^*p* < 0.05 versus the negative control group; ^#^*p* < 0.05 versus UVB-stimulated groups.

Masson's trichrome stains collagen fibers whose density is reduced upon UVB irradiation [9]. Results of Figure 8 confirmed that UVB irradiation induces significant degradation of collagen fibers in the dermis. The uTF was again inactive, and the TFcPGJ_2_ partially reversed the collagen degradation (Figure 8).

### 3.8. TFcPGJ_2_ Reduces Epidermal Thickening Triggered by UVB Irradiation

**Figure 9:**
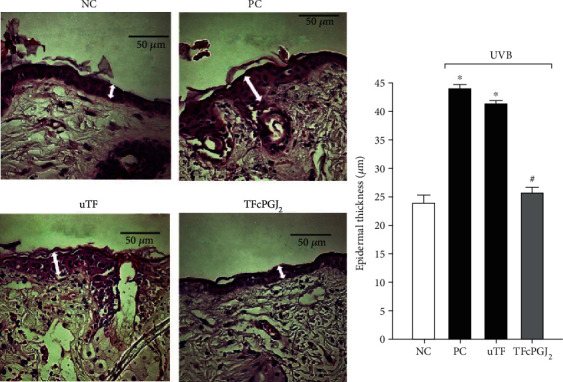
Topical formulation containing 15d-PGJ_2_ (TFcPGJ_2_) inhibits UVB irradiation-induced epidermal thickening. Epidermal thickness (double-edge white arrows) was evaluated using hematoxylin and eosin-stained slices of skin samples collected 12 h after the end of irradiation. Stained slices were examined using light microscopy at 40x magnification. NC: negative control; PC: positive control; uTF: unloaded topical formulation. Bars are means ± SEM of 6 mice per group and are representative of two separate experiments. One-way ANOVA followed by Tukey's test. ^∗^*p* < 0.05 versus the negative control group; ^#^*p* < 0.05 versus UVB-stimulated compared groups.

The thickening of the epidermis is another important tissue alteration caused by UVB irradiation. Epidermal thickening is a result of varied mechanisms that include edema formation and proliferation of the epidermal cells [34]. Both UVB irradiation and UVB irradiation plus uTF induced similar and significant thickening of the epidermis compared to the naive control group. TFcPGJ_2_ reduced the UVB irradiation-triggered thickening of the epidermis (Figure 9).

## 4. Discussion

Sun exposure is essential to, for instance, bone metabolism [35]. On the other hand, excessive sun exposure is a cause of skin cancers [36]. These two opposite situations clearly indicate to us that a balanced relationship with sun exposure must be achieved. In an attempt to find this balance, sun blockers and sunscreens are used. These are effective preventive approaches; however, they are not fully active in bringing sun protection to our skin since they do not counteract skin alterations caused by UVB irradiation but rather focus on preventing the UVB incidence in the skin. The present results demonstrate that a topical formulation containing 15-deoxy-*Δ*^12,14^-prostaglandin J_2_ (TFcPGJ_2_) is active in counteracting both the inflammatory and oxidative skin pathological events known as crucial to induce diseases caused by prolonged and repetitive sun exposure.

The present data deal with skin inflammation and oxidative stress in UVB irradiation and the effect of TFcPGJ_2_. We used an unloaded topical formulation (uTF) as a control that supported that the efficacy of TFcPGJ_2_ is dependent on 15-deoxy-*Δ*^12,14^-prostaglandin J_2_ (15d-PGJ_2_). The selection of this lipid considered that few data has investigated its activity in the skin. In terms of skin cancers, 15d-PGJ_2_ seems to be a useful pharmacological approach to inhibit neoplastic cell proliferation. In terms of inflammation, data show that 15d-PGJ_2_ reduces atopic dermatitis. However, to our knowledge, there is no data on the potential therapeutic effect of 15d-PGJ_2_ in the skin in UVB irradiation inflammation and oxidative stress. Furthermore, there is no evidence that TFcPGJ_2_ is active in that context.

UVB irradiation mechanisms to induce inflammation in the skin involve oxidative stress, cellular activation, and inflammatory enzymes and mediators [9, 11]. It is likely that oxidative stress is the first step in UVB irradiation mechanisms followed by inflammation but with a role of continuous oxidative stress and inflammation depending on the intensity and repetitive stimulation [37]. Chromophore chemical groups react to UVB irradiation producing reactive oxygen species (ROS) [38]. Keratinocytes are skin cells that are majorly affected by UVB irradiation and produce ROS [39]. One side of ROS is related to their tissue injury effects by damaging DNA, lipids, and proteins [11]. The other side is the signaling role of ROS that can activate the transcription factor NF-*κ*B, which is a master regulator of inflammatory genes ranging from inflammatory enzymes to soluble mediators [40]. Cyclooxygenases and lipoxygenases are clinical therapeutic targets to reduce inflammation [41]. These enzymes are activated during varied inflammatory diseases. In UVB irradiation, it is not different, but the significant contribution of ROS to skin disease highlights that ROS are byproducts of the activity of cyclooxygenases and lipoxygenases [42], thus indirectly contributing to inflammation and amplification of oxidative stress. Prostanoids, such as prostaglandin E_2_ (PGE_2_), are produced upon cyclooxygenase activity and are known to orchestrate tissue edema formation and other functions [43]. For instance, the treatment with the selective COX-2 inhibitor celecoxib diminishes skin edema, MPO activity, and the formation of sunburn cells in the skin concomitantly with inhibition of PGE_2_ production [44]. In agreement with that finding in mouse skin, the time course of PGE_2_ production upon UVB irradiation of the skin of healthy humans aligns with the erythema formation [45]. Thus, PGE_2_ exemplifies the contribution of prostanoids to skin inflammation triggered by UVB. Furthermore, ROS themselves such as superoxide anion also cause tissue edema [46]. TFcPGJ_2_ reduced skin edema, which is a classical cardinal sign of inflammation and a disease parameter that when inhibited supports that an anti-inflammatory effect is under observation.

Although cellular recruitment is not a classical cardinal sign of inflammation, this event underlies virtually all inflammatory responses. Cells are recruited to the inflammatory foci to phagocytose, kill pathogens, and clear up cellular debris allowing tissue repair (e.g., neutrophils and macrophages) [47, 48]. Tissue repair also depends on recruited cells (e.g., fibroblasts) that produce the extracellular matrix [49]. We observed the recruitment of neutrophils indirectly by the myeloperoxidase activity and mast cell counts. Neutrophils have a beneficial role during infections, but in sterile diseases such as UVB irradiation, neutrophils contribute to tissue injury by producing MMP-9 [50]. Mast cells have dual functions by acting as recruited cells that enhance inflammation and tissue-resident cells that are the first to be activated and by producing inflammatory mediators that orchestrate the recruitment of, for instance, neutrophils. Mast cells produce IL-6 [51], which has been demonstrated to contribute to the recruitment of neutrophils [52]. Therefore, the effect of TFcPGJ_2_ of inhibiting MMP-9, myeloperoxidase activity, mast cell counts, and IL-6 production aligns to explain how this topical treatment could diminish skin inflammation. In other experimental conditions, PGJ_2_ reduced the expression of MMP-9 and breast cancer MCF-7 cell invasiveness [53], IL-6 production and neutrophil recruitment in gout arthritis [54], and mast cell infiltration in atopic dermatitis [5]. Thus, the present mechanisms of TFcPGJ_2_ line up with previous data on breast cancer cells and skin and articular inflammation.

We observed that TFcPGJ_2_ preserved the skin's endogenous antioxidant repertoire since GSH levels, total sulfhydryls, and catalase activity were kept to basal levels contrasting with their depletion upon UVB irradiation plus uTF administration. ROS production such as of superoxide anion was also brought to basal levels together with the mRNA expression of gp91^phox^ and COX-2. The NADPH oxidase (NOX) family comprises gp91^phox^ (also known as NOX2) isoforms including the NOX1 up to NOX5 and dual oxidases (DUOX) 1 and 2 [55]. Evidence supports a role for NOX1 [56, 57], NOX2 [58, 59], NOX4 [60, 61], and DUOX2 [62] in UVB effects in the skin and skin cells via superoxide anion production. The gp91^phox^ is characteristically expressed by phagocytes such as neutrophils [55], and UVB upregulates its mRNA expression in the skin [59], thus explaining its selection for evaluation in the present study. Furthermore, superoxide anion is a byproduct of COX-2 activity [42]. Thus, the reduction of neutrophil infiltration and gp91^phox^ and COX-2 mRNA expression [29] are together accounted to explain the reduction of superoxide anion production and tissue inflammation. Overall, the results support that TFcPGJ_2_ prevented skin oxidative stress and inflammation, as mentioned above, and led to reduced activity/secretion of MMP-9. This metalloprotease is responsible for extracellular matrix components' degradation [63], which include collagen. TFcPGJ_2_ reduced collagen degradation by 54% and MMP-9 activity by 35%. Diminished ROS production and preservation of endogenous antioxidants might also have contributed to maintaining collagen integrity [9, 64]. Considering TFcPGJ_2_ reduced tissue injury and consequently the need for tissue repair, it is rational that TFcPGJ_2_ treatment had as an outcome a reduction of TGF-*β* production. This interpretation of the indirect effect of TFcPGJ_2_ finds support in previous data demonstrating that PGJ_2_ does not alter TGF-*β* release by fibroblasts stimulated with LPS [65]. In the same experimental condition, 15d-PGJ_2_ reduced IL-6 release by fibroblasts [65] suggesting a modulation of specific cytokine production. On the other hand, a potentiation effect was observed by combining prednisolone with PGJ_2_ against the profibrotic action of TGF-*β* in synovial fibroblasts of osteoarthritis patients. PGJ_2_ enhanced the effect of prednisolone of inhibiting the profibrotic ALK5 (activin receptor-like kinase 5)/Smad2 (mothers against decapentaplegic homolog 2) signaling [66]. Thus, depending on the disease context and drugs concomitantly administrated or even endogenously produced, 15d-PGJ_2_ can also interfere with the profibrotic actions of TGF-*β*.

Superoxide anion seems to be an important ROS in the induction of keratinocyte apoptosis forming sunburn cells. In fact, treatment with superoxide dismutase that metabolizes superoxide anion to molecular oxygen and H_2_O_2_ prevents the formation of other free radicals and the formation of sunburn cells by UVB irradiation [67]. The catalase converts H_2_O_2_ in molecular oxygen and H_2_O, thus preventing the formation of hydroxyl that would occur via the Fenton reaction [68]. GSH depletion is a consequence of the high production of ROS, and in agreement with that, GSH inhibition enhances sunburn cell formation [69]. Considering these mechanisms described for sunburn cell formation and that TFcPGJ_2_ prevented superoxide anion production and GSH depletion, it is likely that modulating oxidative stress is a mechanism of TFcPGJ_2_ to reduce sunburn formation in the epidermis. Another characteristic pathological skin alteration is the thickening of the epidermis. This process may involve varied mechanisms that induce the proliferation of epidermal cells as well as keratinocytes, recruitment of immune cells, and exudation due to inflammation. Superoxide anion is an example of a mediator that induces edema [70].

**Figure 10:**
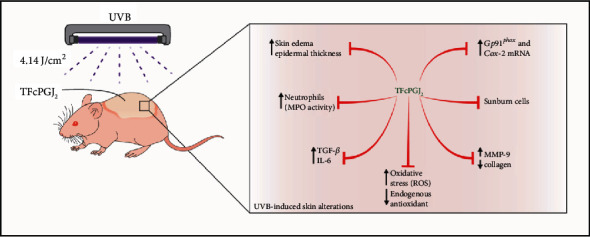
Schematic representation of the effects of topical formulation containing 15d-PGJ_2_ (TFcPGJ_2_) against skin alterations upon UVB irradiation. UVB causes inflammatory disease signs such as skin edema as well as pathological alterations in the skin including sunburn cell formation, an increase of mast cell counts, thickening of the epidermis, and collagen degradation. These phenomena are explained by the induction of neutrophil recruitment observed as myeloperoxidase (MPO) activity, metalloproteinase-9 (MMP-9) activity, reactive oxygen species (ROS) production, depletion of endogenous antioxidants, enhanced mRNA expression of enzymes involved in the production of ROS and inflammatory mediators such as gp91^phox^ and cyclooxygenase-2 (COX-2), and cytokine production (TGF-*β* and IL-6). All these UVB pathological modifications in the skin were inhibited by TFcPGJ_2_ and unaltered by an unloaded topical formulation (uTF—not mentioned in the schematic figure).

In conclusion, we have shown, to our knowledge, for the first time that 15d-PGJ_2_ reduces UVB irradiation pathological inflammatory and oxidative alterations in the skin. 15d-PGJ_2_ can be administrated using a topical nonionic cream as a vehicle to successfully achieve its therapeutic effects directly on the target tissue.  Figure 10 summarizes the findings of the present study.

## Figures and Tables

**Table 1:** Primer sequences.

Gene	Sense primer	Antisense primer
*Gp91^phox^*	AGCTATGAGGTGGTGATGTTAGTGG	CACAATATTTGTACCAGACAGACTTGAG
*Cox*-*2*	*GTGGAAAAACCTCGTCCAGA*	*GCTCGGCTTCCAGTATTGAG*
*Gapdh*	CATACCAGGAAATGAGCTTG	ATGACATCAAGAAGGTGGTG

## Data Availability

The data used to support the findings of this study are included within the article.
